# Central and Peripheral Characterization of Key Kynurenine Pathway Metabolites in Mexican Patients with Multiple Sclerosis: An Exploratory Study

**DOI:** 10.3390/ph19030513

**Published:** 2026-03-21

**Authors:** Pablo Arturo Acosta Mendez, Graciela Ordoñez, Karla F. Meza-Sosa, Tonali Blanco Ayala, Daniela Ramirez Ortega, Gonzalo Pérez de la Cruz, Dinora F. González Esquivel, Teresita Corona, José Flores Rivera, Verónica Rivas, Paul Carrillo Mora, Carmen Aláez-Verson, Korrapati V. Sathyasaikumar, Saúl Gomez-Manzo, Aleli Salazar, Benjamin Pineda, Verónica Pérez de la Cruz

**Affiliations:** 1 Neurobiochemistry and Behavior Laboratory, National Institute of Neurology and Neurosurgery “Manuel Velasco Suárez”, Mexico City 14269, Mexico; contacto@drpabloamendez.com (P.A.A.M.); karla.meza@innn.edu.mx (K.F.M.-S.); tblanco@innn.edu.mx (T.B.A.); danielaramirez@innn.edu.mx (D.R.O.); dinora.gonzalez@innn.edu.mx (D.F.G.E.); 2 Neuroimmunology Department, National Institute of Neurology and Neurosurgery “Manuel Velasco Suárez”, Mexico City 14269, Mexico; graciela.ordonez@innn.edu.mx (G.O.); aleli.salazar@innn.edu.mx (A.S.); 3 Department of Mathematics, Faculty of Sciences, Universidad Nacional Autónoma de México (UNAM), Mexico City 04510, Mexico; gonzalo.perez@ciencias.unam.mx; 4 Clinical Laboratory of Neurodegenerative Diseases, National Institute of Neurology and Neurosurgery “Manuel Velasco Suárez”, Mexico City 14269, Mexico; coronav@unam.mx; 5 Multiple Sclerosis and Demyelinating Disorders Clinic, National Institute of Neurology and Neurosurgery “Manuel Velasco Suárez”, Mexico City 14269, Mexico; jflores@innn.edu.mx (J.F.R.); veronica.rivas@innn.edu.mx (V.R.); 6 Clinical Neurosciences Division, National Institute of Rehabilitation “Luis Guillermo Ibarra Ibarra”, Mexico City 14389, Mexico; pcarrillo@inr.gob.mx; 7 Laboratory of Genomic Diagnostics, National Institute of Genomic Medicine, Mexico City 14610, Mexico; calaez@inmegen.gob.mx; 8 Maryland Psychiatric Research Center, Department of Psychiatry, University of Maryland School of Medicine, Baltimore, MD 21228, USA; saikumar@som.umaryland.edu; 9 Laboratorio de Bioquímica Genética, Instituto Nacional de Pediatría, Secretaría de Salud, Mexico City 04530, Mexico; saulmanzo@ciencias.unam.mx

**Keywords:** quinolinic acid, multiple sclerosis, kynurenine pathway

## Abstract

**Background/Objectives**: Multiple Sclerosis (MS) is a chronic immune-mediated disorder characterized by neuroinflammation and neurodegeneration. Increasing evidence implies the kynurenine pathway (KP) in the MS pathophysiology; however, data from Mexican populations are lacking. This exploratory study aimed to characterize central and circulating KP metabolites in Mexican patients with MS and to investigate potential genetic variants in KP-related genes. **Methods**: Serum concentrations of kynurenic acid (KYNA) and 3-hydroxykynurenine (3-HK), as well as cerebrospinal fluid (CSF) levels of KYNA, quinolinic acid (QUIN), interleukin-4 (IL-4), and interleukin-6 (IL-6), were determined in treatment-naïve relapsing-remitting MS (RRMS), primary progressive MS (PPMS), and treated PMS patients. Serum levels were compared with those of healthy controls, and CSF findings contrasted with those of non-MS neurological patients and individuals with neurocysticercosis (NCC). Public whole-exome datasets were analyzed for variants in KP-related genes, and target exome sequencing was performed in three Mexican patients with MS. **Results**: Serum concentrations of KYNA and 3-HK were decreased in MS patients compared with healthy controls. CSF KYNA and QUIN levels did not differ significantly among MS subtypes or the non-MS neurological group, but they were lower than those observed in NCC. IL-4 and IL-6 were detectable in MS CSF samples, supporting the presence of intrathecal inflammation. Genetic and bioinformatic analyses identified variants in genes encoding KP enzymes in both public MS datasets and in Mexican patients with MS. **Conclusions**: These findings indicate an altered KP metabolism in Mexican MS patients, particularly during the relapse phase, and suggest a possible contribution of genetic variability. Further large-scale studies are needed to confirm these observations and to determine the functional implications of KP-related genetic variants in MS.

## 1. Introduction

Multiple Sclerosis (MS) is a chronic autoimmune, inflammatory, and demyelinating disease that affects the Central Nervous System (CNS). It is characterized by damage occurring at different times and locations within the CNS, leading to both axonal and myelin damage. MS manifests through episodes of neurological dysfunction, with varying degrees of recovery, disease progression, and disability. The clinical course of MS includes relapsing-remitting MS (RRMS), which is the most common clinical type of MS, characterized by alternating periods of disease activity and inflammation (relapses) and remission (periods of complete or partial clinical recovery). The progressive form of MS (PMS) is marked by a gradual accumulation of disability that progresses independently of relapses [1]. Approximately 2.3 million people have been diagnosed with MS worldwide. In Mexico, the disease affects around 20,000 individuals, making it the second leading cause of disability among the working-age population. Epidemiological and genetic ancestry studies indicate that the average age of MS’s onset, prevalence, and clinical phenotypes vary between the Caucasian and the Latino populations. Latino patients often present MS at a younger age, exhibit more severe visual or spinal cord symptoms, and experience faster disability progression. A comparative study in 2021 found that the Hispanic population was more likely to have a lower survival ratio than Caucasians [2].

The pathogenesis of MS remains incompletely understood; however, it is well established that the disease involves a complex interplay of aberrant immune response, demyelination, neuroaxonal degeneration, and synaptic loss, among other contributing factors. In recent years, increasing attention has been paid to the dysregulation of the kynurenine pathway (KP) as a potential player in MS pathophysiology, as it is believed to result from chronic inflammatory stimulation of the pathway [3,4,5]. The KP constitutes the primary cellular route of tryptophan (Trp) catabolism, primarily to support *de novo* synthesis of NAD+. Along this pathway, several bioactive metabolites are generated, each with distinct immunomodulatory, redox, and neuroactive properties. Trp is initially cleaved by either the tryptophan dioxygenase (TDO) or the indoleamine 2,3-dioxygenase (IDO), resulting in the formation of L-kynurenine (KYN), a central intermediate in the KP. KYN serves as a substrate for three distinct enzymatic branches: (1) the kynurenine aminotransferases (KATs), which constitute the short branch of the pathway and convert KYN into kynurenic acid (KYNA), a neuromodulator metabolite; (2) the kynurenine monooxygenase (KMO), representing the long branch, which produces 3-hydroxykynurenine (3-HK), a redox-active metabolite; and (3) the kynureninase (KYNU), which catalyzes the conversion of KYN into anthranilic acid (AA). AA can be further hydroxylated to 3-hydroxyanthranilic acid (3-HANA), either by the anthranilate 3-hydroxylase or by KYNU acting on 3-HK [6,7]. Following the KP, 3-HANA serves as a precursor to quinolinic acid (QUIN) formation, a neuroactive metabolite that is subsequently used to synthesize nicotinamide adenine dinucleotide (NAD+), the final product of the KP and an essential coenzyme in cellular energy metabolism. While KYNA and QUIN are neuroactive and redox-active metabolites that do not cross the blood–brain barrier (BBB), other intermediates, such as KYN, 3-HK, and 3-HANA, can cross the BBB and mediate peripheral–central interactions [8,9,10]. Dysregulation of KP metabolite levels has been associated with several neurodegenerative and psychiatric conditions, including Alzheimer’s disease, Parkinson’s disease, schizophrenia, and MS [3,11,12,13,14,15,16,17]. Importantly, KP activity is strongly influenced by inflammatory and oxidative conditions, and in turn, KP metabolites can reinforce these conditions, forming a feedback loop that may exacerbate disease onset and progression [18].

In this context, MS patients have been shown to exhibit elevated basal levels of cytokines, including interleukin-4 (IL-4), interleukin-6 (IL-6), interferon gamma (INF-γ), and tumor necrosis factor-alpha (TNF-α) [19,20]. It has been proposed that this inflammatory environment indirectly promotes KP dysregulation, as these cytokines are potent inducers of IDO1, thereby enhancing KP activation in both peripheral immune cells and resident brain immune cellular populations. In this line, altered production of KP metabolites, together with changes in IDO expression and enzymatic activity, have been described during relapse phases in patients with RRMS [3,21]. Studies have reported reduced plasma or serum levels of Trp, KYN, 3-HK, and KYNA in patients with RRMS, while 3-HANA or AA are increased [22,23]. At the central level, cerebrospinal fluid (CSF) analyses have shown a dynamic regulation of KP metabolites, with KYNA concentrations increasing during relapse and declining during remission [5,24,25]. Notably, QUIN levels, as well as the QUIN/KYNA ratio, are significantly elevated in RRMS patients during relapse phases [5]. Conversely, other studies have reported increased activity of KATI and KATII in red blood cells from MS patients, which is associated with higher plasma KYNA levels [26]. These seemingly contradictory findings likely reflect heterogeneity in study design, including differences in disease stage at the time of sampling, the type of biological samples used, the exposure to disease-modifying therapies, and the use of anti-inflammatory drugs (corticosteroids). In this exploratory study, we aimed to determine serum levels of 3-HK and KYNA, as well as CSF levels of KYNA and QUIN, in a cohort of Mexican patients with MS. This study included treatment-naïve patients with RRMS, patients with primary progressive MS (PPMS), and PPMS patients receiving immunomodulatory therapy. Serum metabolite levels were compared with those from healthy individuals; similarly, CSF metabolite concentrations were contrasted with samples from patients presenting other inflammatory or neurological conditions that warranted CSF sampling in the emergency setting. Additionally, to explore potential genetic contributions to KP’s dysregulation in the MS context, we identified the presence of genetic variants in KP-related genes by analyzing publicly available whole-exome sequencing (WES) datasets from two independent MS patient cohorts, and from three Mexican MS patients, revealing potential new therapeutic molecular targets to better understand the pathophysiology of MS.

## 2. Results

### 2.1. Demographic Characteristics of Mexican Healthy and MS Individuals

The study sample comprised a total of 50 patients diagnosed with MS, including 25 individuals with RRMS (50%), 16 with PPMS (32%), and 9 patients with PPMS receiving disease-modifying treatment (PMSt; 18%), all of them in the relapse phase. For peripheral analyses, serum samples from MS patients were compared to those obtained from 20 healthy participants (70% women) serving as controls. CSF metabolite levels were contrasted with samples from two outgroups: five patients diagnosed with neurocysticercosis (NCC) and twelve patients presenting other neurological conditions (Non-MS) who were evaluated at the emergency room and underwent CSF sampling as part of their clinical assessment.

Demographic and clinical characteristics of the sample studied are listed in Table 1. A predominance of women was observed in the RRMS and PPMS groups (72% and 62.5%, respectively), a distribution comparable to that of the healthy control (HC) group (70%) and the neurological group without MS (83.3%). The mean age at diagnosis among MS subtypes was 29.7 ± 10 years for RRMS, 35.1 ± 5.3 years for PPMS, and 29.2 ± 7.8 years for PMSt. No statistically significant differences were detected between subgroups and the HC group. In contrast, the non-MS group was significantly older than the control group (*p* = 0.004). Neurological disability was assessed using the Expanded Disability Status Scale (EDSS) at the time of biological sample collection (serum or CSF). EDSS scores were available for 19 patients in the RRMS group, for 5 in the PPMS group, and for 6 in the PMSt group. The mean EDSS score was 1.9 ± 0.9 for RRMS, 5.5 ± 2.6 for PPMS, and 7.1 ± 1.7 for PMSt patients. Notably, both the RRMS and PPMS groups were treatment-naïve at the time of sampling, and samples from RRMS patients were obtained during the clinical relapse phase.

### 2.2. Imbalance of KP Metabolite Production in the Periphery and the CSF of Mexican MS Patients

Because biological samples were obtained from an existing biobank, the available serum and CSF were insufficient to quantify the full spectrum of KP metabolites in all patient-derived samples. Therefore, peripheral analyses were limited to determine KYNA and 3-HK levels, as these are representative metabolites of the short and long arms of the KP, respectively. Serum concentrations were compared across MS subtypes and healthy controls to explore the potential shifts in KP activity.

**Figure 1:**
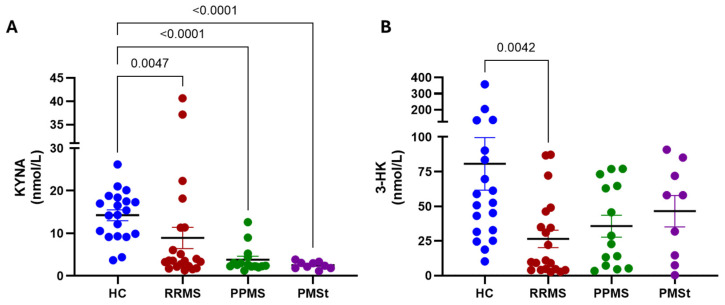
Level of circulating kynurenine pathway metabolites in Mexican MS patients. (**A**) Kynurenic acid (KYNA) and (**B**) 3-hydroxykynurenine (3-HK) concentrations in serum of relapsing-remitting Multiple Sclerosis (RRMS), primary progressive MS (PPMS), and PMS under treatment (PMSt) patients compared to healthy controls (HC). Data are represented as mean ± SEM. *p*-values were obtained by performing the Kruskal–Wallis test, followed by pairwise comparison based on Dunn’s tests.

As shown in Figure 1A, all MS subtypes showed significantly lower serum KYNA levels compared to the healthy control group (RRMS: 8.9 ± 2.5 nM; PPMS: 3.8 ± 0.8 nM; PMSt: 2.5 ± 0.3 nM compared to HC: 14.3 ± 1.3 nM). Regarding 3-HK (Figure 1B), a significant reduction was observed only in the RRMS group compared to the HC group (26.5 ± 6.3 nM vs. 80.61 ± 19 nM). Although the PPMS and PMSt groups also showed lower mean 3-HK concentrations (35.7 ± 8 nM and 46.5 ± 11.4 nM, respectively), these differences did not reach statistical significance. When the 3-HK/KYNA ratio was calculated, a non-significant trend toward higher values was observed across all MS subtypes compared to participants in the HC group (RRMS: 8.2 ± 2.5 nM; PPMS: 12.2 ± 4.2 nM; PMSt 21.6 ± 7.7 nM vs. HC: 5.1 ± 0.9 nM).

**Figure 2:**
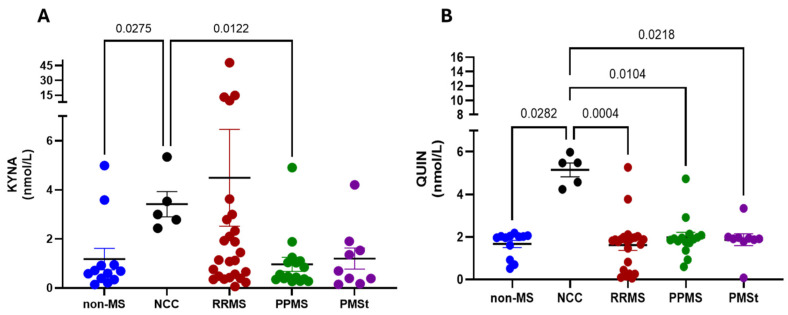
KP metabolite levels in CSF of Mexican MS patients. CSF concentration of (**A**) kynurenic acid (KYNA) and (**B**) quinolinic acid (QUIN) in Mexican non-MS neurological condition (non-MS) patients, patients with neurocysticercosis (NCC), relapsing-remitting Multiple Sclerosis (RRMS) patients, primary progressive MS (PPMS) patients, and patients with PMS that are under treatment (PMSt). Data are represented as mean ± SEM. *p*-values were obtained using the Kruskal–Wallis test, followed by pairwise comparisons based on Dunn’s tests.

CSF concentrations of KYNA and QUIN were subsequently determined in all MS subtypes and compared with two reference outgroups: patients with non-MS neurological conditions who underwent lumbar puncture in the emergency room, and patients diagnosed with NCC. Among all the analyzed groups, individuals with NCC exhibited the highest levels of both KYNA and QUIN in CSF (Figure 2). Regarding KYNA, no statistical differences were detected between MS subtypes and the non-MS neurological group (RRMS: 4.5 ± 9.8 nM; PPMS: 0.96 ± 0.3 nM; PMSt: 1.2 ± 0.4 nM vs. non-MS: 1.2 ± 0.4 nM). However, KYNA concentrations were significantly lower in the non-MS and PPMS groups compared to patients with NCC (3.4 ± 0.5 nM). Similarly, QUIN concentration did not differ significantly among MS subtypes and the non-MS neurological group (RRMS: 1.6 ± 0.3 nM; PPMS: 2.0 ± 0.2 nM; PMSt: 1.9 ± 0.3 nM vs. non-MS: 1.7 ± 0.2 nM). In contrast, QUIN levels were significantly elevated in the NCC group (5.1 ± 0.3 nM). When the QUIN/KYNA ratio was calculated, no statistically significant differences were detected between groups (non-MS: 3.8 ± 1.3 nM, NCC: 1.6 ± 0.2 nM; RRMS: 3.7 ± 1.4 nM; PPMS: 3.8 ± 0.8 nM; PMSt: 5.5 ± 2.4 nM).

**Figure 3:**
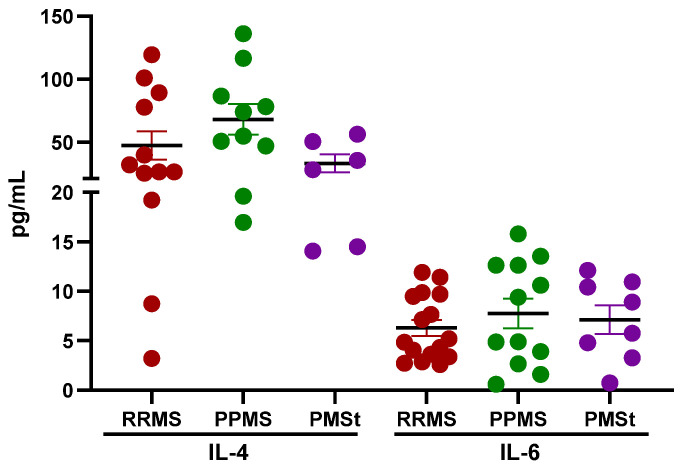
IL-4 and IL-6 concentrations in CSF of Mexican MS patients. Concentrations of interleukin 4 (IL-4) and interleukin 6 (IL-6) in CSF derived from relapsing-remitting Multiple Sclerosis (RRMS) patients, primary progressive MS (PPMS) patients, and PMS patients that are receiving treatment (PMSt). Data are represented as mean ± SEM. *p*-values were obtained using the Kruskal–Wallis test, followed by pairwise comparisons based on Dunn’s test.

To further characterize the inflammatory milieu within the CNS, the concentrations of IL-4 and IL-6 were determined in CSF samples from MS patients. IL-4 levels were determined from 12 RRMS patients, 10 PPMS patients, and 6 PMSt patients. As shown in Figure 3, no statistically significant differences were detected among MS subtypes (RRMS: 47.3 ± 11.2 pmol/mL; PPMS: 68.0 ± 12.2 pmol/mL, and PMSt: 33.1 ± 7.2 pmol/mL) for this cytokine. Concentration of IL-6 was determined in CSF samples obtained from 16 RRMS patients, 12 PPMS patients, and 8 PMSt patients. Similarly to IL-4, no significant differences were observed among groups for IL-6 (RRMS: 6.3 ± 0.8 pmol/mL; PPMS: 7.8 ± 1.5 pmol/mL; PMSt: 7.1 ± 1.5 pmol/mL), as shown in Figure 3. Although intergroup differences were not statistically significant, the detection of both IL-4 and IL-6 in CSF samples supports the presence of an active inflammatory process within the CNS. Moreover, the concurrent identification of an anti-inflammatory cytokine (IL-4) and the proinflammatory mediator, IL-6, may reflect an ongoing immune regulation, suggesting a dynamic balance between inflammatory and compensatory anti-inflammatory immune mechanisms in MS patients.

### 2.3. Correlation Between KP Metabolites’ Levels, Inflammatory Markers, and Disability in MS Patients

**Figure 4:**
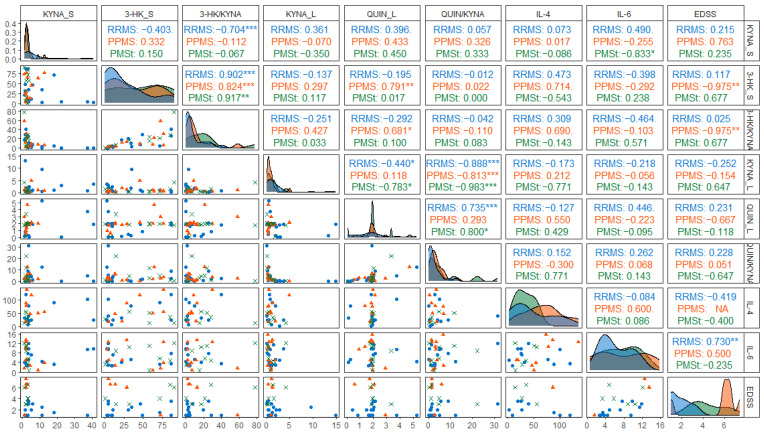
Correlation matrix showing pairwise associations among the levels of serum KYNA and 3-HK, CSF concentrations of KYNA, QUIN, IL-4, and IL-6, and Expanded Disability Status Scale (EDSS) scores. The lower-left triangular matrix contains scatter plots, while the upper-right triangular matrix displays the Spearman’s coefficient (r) for each patient’s group (RRMS, PPMS, PMSt). *p*-values are denoted using the following significance levels: for *p* < 0.1, * for *p* < 0.05, ** for *p* < 0.01, and *** for *p* < 0.0001.

Despite the limited availability of biological samples and incomplete EDSS data in some cases, exploratory correlation analyses were conducted to examine the potential association between KP metabolite levels, the presence of inflammatory markers, and neurological disability in Mexican MS patients (Figure 4). In patients with PPMS, the serum 3-HK/KYNA ratio showed a positive correlation with QUIN concentrations in CSF. In the same group, both serum 3-HK levels and the 3-HK/KYNA ratio were negatively correlated with EDSS scores. Additionally, in RRMS patients, CSF IL-6 levels demonstrated a positive correlation with EDSS. These associations should be cautiously interpreted, given the reduced sample size and the exploratory nature of the analysis.

### 2.4. Identification of Genetic Variants in KP Metabolism-Related Genes in Independent Familial MS Cohorts

Due to the observed alterations in KP metabolite levels in MS patients, we decided to explore whether genetic variants within genes related to Trp metabolism could be identified in MS patients. For this, publicly available whole-exome sequencing (WES) data from two independent MS cohorts were selected and retrieved from the Sequence Read Archive (SRA) repository. The raw WES data consisted of 14 FASTAQ files, which included data from eight individuals diagnosed with MS and six unaffected relatives (all 14 individuals belonging to four independent families detailed in Materials and Methods). After the WES data were assessed for quality control and aligned to the reference human genome, genetic variants of single nucleotides were searched only in those genes encoding proteins involved in the KP, and then, detected variants were also evaluated in healthy relatives to determine their genetic segregation patterns and assess their potential enrichment only in MS-affected individuals (Table 2). Among the identified variants, two missense substitutions exhibited low allele frequencies in the general population according to gnomAD v.4.0 and were classified as variants of uncertain significance (VUS). One of these variants (NM_003486.7[SLC7A5]:c.920C>T) has not been conclusively linked to a defined clinical phenotype, although previous reports have associated it with the autism spectrum disorder [27]. The second variant, NM_015836.4[WARS2]:c.119G>A, has been described as being associated with a neurodevelopmental disorder exhibiting an autosomal recessive inheritance pattern [28]. The remaining variants in KP-related genes were classified as benign variants (VB) based on the American College of Medical Genetics and Genomics (ACMG) guidelines 2015.

Following the identification of variants in genes encoding KP enzymes in publicly available MS WES datasets, we sought to determine whether similar genetic alterations could be detected in Mexican MS patients. For this end, WES was performed in samples obtained from three unrelated Mexican MS individuals (two with RRMS and one with PPMS). Then, a search for genetic variants was subsequently conducted, focusing specifically on genes involved in Trp metabolism. This exploratory analysis identified multiple variants of single nucleotides in genes encoding key enzymes of the KP, including *GOT2* (KATIV), *IDO1*, *IDO2*, and *QPRT* (Table 3). Several variants were shared across the analyzed patients, notably GOT2:c.1037T>G (p.Val346Gly), HAAO:c.109A>G (p.Ile37Val), and QPRT:c.583A>G (p.Thr195Ala), which were observed in a homozygous form in at least two of the three individuals. Moreover, additional variants were detected in the *IDO2* gene, including a nonsense one (c.1077T>A; p.Tyr359*) in one of the two RRMS patients, and an homozygous missense variant (c.742C>T; p.Arg248Trp) in the other patient; while in the *IDO1* gene the variant c.425C>T; p.Pro142Leu was detected in the PPMS patient.

## 3. Discussion

In recent years, there has been growing evidence supporting the role of the KP in the pathogenesis of MS. Given the chronic inflammatory nature of MS, it has been proposed that proinflammatory signals such as IL-6, INF-γ, and TNF-α can enhance the activity of KP enzymes such as IDO1 and KMO. Upregulation of these enzymes may shift Trp metabolism toward the generation of downstream metabolites, including QUIN, at the expense of KYNA, thereby altering the balance of this pathway [19,20]. BBB disruption, a recognized feature of active MS lesions, further amplifies this process. Increased BBB permeability facilitates the infiltration of peripheral immune cells capable of expressing KP enzymes; therefore, infiltrating macrophages and resident microglia may enhance local QUIN production under inflammatory stimulation [31,32,33]. In this context, some groups have found a relationship between the serum QUIN/KYNA ratio, disease severity, and cognitive decline in MS [32,34]. A recent systematic review has consolidated these findings, highlighting consistent alterations of KP metabolites across different MS populations [18].

In this exploratory study, we decided to characterize the KP profile in a Mexican cohort of MS patients by integrating clinical, biochemical, and genetic analyses. Our findings are broadly consistent with previous reports describing KP dysregulation in MS patients. Specifically, the reduction in circulating KYNA and 3-HK levels observed across MS subtypes in our cohort is consistent with studies suggesting altered KP flux under chronic inflammatory conditions [22]. Although 3-HK concentration decreased, the calculated 3-HK/KYNA ratio showed a non-significant trend toward higher values in MS patients compared with those of controls. While exploratory and limited by sample size, this trend may reflect a relative shift toward the KMO branch of the pathway. Since 3-HK can cross the BBB, it may contribute to central QUIN production. Notably, reduced circulating 3-HK levels have been associated with increased microglial activation in normal-appearing white matter and the thalamus, as well as with greater clinical disability in MS patients [35]. In our cohort, serum 3-HK levels and the 3-HK/KYNA ratio were negatively correlated with the EDSS scores in patients with PPMS. Although this finding may appear counterintuitive in light of the proposed neurotoxic role of the KMO branch, it should be interpreted with caution, given the small size of the PPMS subgroup. Otherwise, in this same PPMS subgroup, it is interesting to note that a positive correlation was found between serum levels of 3-HK and QUIN in CSF, which would suggest a particular regulation of the KMO branch at the central level through the exogenous supply of 3-HK. The potential role of 3-HK in regulating MS pathogenesis through microglial activation, including local regulation of the proinflammatory brain environment, should be further explored by considering future comparisons between serum vs. CSF levels of 3-HK.

When KYNA and QUIN levels were determined in CSF, only modest differences were observed between the MS group and the neurological group without MS. In contrast, patients with NCC exhibited markedly higher concentrations of both metabolites. This pattern is biologically plausible, as NCC is typically associated with intense inflammatory responses within the CNS, which may drive robust activation of the KP and favor the accumulation of downstream metabolites [36]. Additionally, the reason why no significant difference was observed between the MS groups and patients with no-MS pathologies may be because the most frequent indication for CSF extraction is neuroinfectious, which can show a pattern of acute inflammation similar to that of patients with MS [37]. Importantly, although CSF KP metabolite levels in MS patients did not reach the magnitude observed in NCC, the presence of IL-4 and IL-6 in the CSF indicates that an inflammatory environment is persistent in all MS subgroups. Under physiological conditions, cytokine concentrations in CSF are low or undetectable; therefore, their detection supports the existence of ongoing intrathecal immune activation. It is important to mention that most KP-MS studies compare CSF KP metabolite levels to non-inflammatory neurological controls, but we were unable to do so given the ethical limitations involved in obtaining CSF from healthy individuals for an exploratory study. Moreover, the fact that these results represent single-time-point measurements must be considered, as this prevents us from making inferences about disease progression, causality, or temporal KP dynamics. However, we must not overlook the fact that the inclusion of treatment-naïve patients from different stages of MS is rarely reported. However, once again, it is possible that the absence of differences observed between the groups with vs. without treatment is because the patients with treatment were actually patients with active disease at the time of CSF sampling (treatment failure). Thus, including this type of sample in future studies could provide better insights into the onset and the pathogenesis of the disease. Future studies incorporating a more detailed analysis of an expanded panel of kynurenines will be necessary to determine whether the observed alterations reflect a global activation of the KP or a selective and local regulation of the enzymes involved.

Following the biochemical characterization of KP metabolites in both serum and CSF, our findings can be interpreted within the broader context of inflammatory activation during MS relapse. Acute inflammatory episodes are known to stimulate enzymes of the KP, thereby enhancing the production of downstream metabolites, evidence that has also been described in previous clinical studies [5,24]. In particular, increased *IDO1* mRNA expression has been reported during the relapse in MS patients, supporting the concept of inflammation-driven activation of Trp catabolism [21,38]. The resulting increase in KP flux during symptomatic periods may consequently contribute to neurochemical imbalance. In this line, KYNA plays a modulatory role at glutamatergic and cholinergic synapses through its antagonistic effects on NMDA and α7-nicotinic receptors. Alterations in KYNA levels may therefore influence glutamatergic, cholinergic, and dopaminergic neurotransmission, potentially affecting cognitive and motor functions, frequently impaired in MS patients. In parallel, dysregulated production of QUIN, particularly by infiltrating macrophages and activated microglia, may exert direct neurotoxic effects. Experimental studies have demonstrated that elevated QUIN levels can promote excitotoxicity and apoptosis in oligodendrocytes as well as in neurons and astrocytes [39,40]. Moreover, QUIN has been shown to inhibit glutamine synthetase activity, thereby impairing the conversion of glutamate to glutamine and disrupting the astrocyte–neuron glutamate–glutamine cycle [41]. This disturbance may contribute to extracellular glutamate accumulation and excitotoxic damage. Additional studies indicate that excessive QUIN can induce mitochondrial dysfunction, promote oxidative stress, trigger hyperphosphorylation of cytoskeletal proteins, and destabilize neuronal architecture [42,43,44]. Collectively, these mechanisms provide a plausible link between inflammation-driven KP activation and neurodegenerative processes in MS.

To our knowledge, genetic variation in genes encoding KP enzymes has been minimally explored in MS patients, limiting direct comparison with prior clinical studies (see [45] for a review). Most existing works focused on analyzing the expression or activity of the KP enzymes and not on the identification of rare genetic variants [45]. The results obtained from this targeted analysis, focusing specifically on genes involved in Trp catabolism, identified multiple variants in genes encoding key enzymes of the KP, with several variants shared across MS patients, and observed in a homozygous form in at least two individuals. Additional variants detected in *IDO1* and *IDO2* genes should be explored in depth to clarify whether they compromise their enzymatic activity and, therefore, the generation of metabolites downstream of the KP. Given the limited number of Mexican MS sequenced individuals and the absence of functional validation, the genetic analyses included here were exploratory and aimed at identifying candidate variants potentially associated with KP metabolism rather than establishing genetic causality.

Despite its exploratory nature, this study presents several important strengths. First, it provides one of the first characterizations of the KP metabolism in a Mexican MS cohort, addressing a significant gap in historically underrepresented populations in MS’s biomarker research. Second, the simultaneous assessment of peripheral and CSF KP metabolite levels enables the evaluation of central–peripheral metabolic relationships, an aspect that is rarely examined in clinical studies due to the limited availability of CSF samples. Third, the inclusion of treatment-naïve patients across distinct MS phenotypes allows preliminary exploration of KP alterations across different stages of MS disease biology. Finally, the integration of metabolite measurements, inflammatory markers, clinical disability assessment, and exploratory genetic analysis offers a multidimensional framework that may guide future mechanistic investigations of KP dysregulation in MS, particularly those related to the pathogenesis of the disease.

This study has several limitations that should be considered when interpreting the results. First, the small sample size may reduce the statistical power to detect real differences among groups, increasing the probability of obtaining non-significant results even when biologically relevant effects may be present. Second, due to the limited volumes of available samples from the biobank, we were unable to quantify the full spectrum of KP metabolites in both serum and CSF. Because the activity of KP is highly influenced by the cellular and inflammatory environment, the interpretation based on a restricted set of metabolites (KYNA and 3-HK in serum and QUIN in CSF) should be approached cautiously when inferring shifts between the different metabolic branches of the pathway. Third, although comparator groups were included for CSF analyses, the limited availability of well-matched controls restricted the ability to determine whether the observed metabolite levels are specific to MS or represent a more general inflammatory response. Finally, the genetic analysis was exploratory and involved a very small number of patients; therefore, the detected variants cannot be interpreted as causal and, as such, require confirmation in larger cohorts as well as functional validation. Future studies including larger populations, more comprehensive metabolite profiling, and appropriate control groups will be necessary to confirm and extend these observations. Accordingly, the present findings should be considered as hypothesis-generating and as a starting point for further investigations of KP dysregulation in MS.

## 4. Materials and Methods

### 4.1. Study Population

Mexican patients with a confirmed diagnosis of MS were identified through the biobank of the National Institute of Neurology and Neurosurgery Manuel Velasco Suárez (INNNMVS), Mexico City. Stored serum and cerebrospinal fluid (CSF) samples were retrieved for the quantification of KP metabolites. The studied cohort comprised 25 patients with RRMS, 16 patients with PPMS, and 9 patients with PMS receiving treatment (PMSt). Demographic and clinical information were obtained from institutional medical records. Neurological disability was evaluated using the Expanded Disability Status Scale (EDSS), as previously assessed by an experienced neurologist. For this study, the EDSS score recorded at the time of lumbar puncture was considered. In RRMS and PPMS patients, this corresponds to the score at diagnosis, when CSF sampling was performed. In contrast, for PMSt patients, the EDSS value reflected their clinical status at the time of reassessment due to insufficient therapeutic response, when a subsequent CSF sample was obtained. This study was reviewed and approved by both the Research and the Ethics Committees of the INNNMVS (approval number 121/22). All procedures were conducted in accordance with the Declaration of Helsinki principles.

The healthy control (HC) group consisted of 20 volunteers aged 20–45 years, matched to the MS cohort by age and sex. None of the control participants had a history of an autoimmune, inflammatory, or neurological disorder at the time of sampling. For CSF comparison, two additional reference outgroups were included: the first one consisted of 5 patients diagnosed with neurocysticercosis (NCC), selected to represent a condition characterized by marked CNS inflammation; the second group included 9 patients presenting a non-MS neurological condition that underwent lumbar puncture for CSF sampling. These two groups allowed the assessment of KP metabolite levels in MS in both highly inflammatory and non-demyelinating neurological contexts.

### 4.2. Kynurenic Acid Determination in Serum and CSF

Serum and CSF samples were deproteinized by mixing 6% perchloric acid in a 1:2 *v/v* ratio, then centrifuged at 14,000× *g* for 10 min. KYNA detection was performed using a fluorescence detector (RF-20Axs, Shimadzu, Tokyo, Japan), with an excitation/emission wavelength of 344/398 nm. The mobile phase comprised 250 mM zinc acetate, 50 mM sodium acetate, and 2% acetonitrile (pH 6.2), delivered at 0.5 mL/min through a reverse-phase C18 column (Nexcol C18, 5 µm, 50 mm × 3.0 mm; Shimadzu, Tokyo, Japan) under isocratic conditions. Retention times were approximately 7 min.

### 4.3. Serum 3-HK Determination

For 3-HK measurement in serum, samples were processed as mentioned above; 100 µL of the supernatant was injected into an Adsorbosphere Catecholamine C18 reverse-phase column (3 µm, 4.6 mm × 100 mm; Fisher Scientific, Hampton, NH, USA). The mobile phase consisted of 0.27 mM EDTA, 8.9 mM heptane sulfonic acid, 9% triethylamine, 0.59% phosphoric acid, and 3% acetonitrile, delivered at 0.4 mL/min. In the eluate, 3-HK was detected using an LC-4C electrochemical detector (BASi, West Lafayette, IN, USA), operated at an oxidation voltage of 0.5 V, a sensitivity range of 1.0 nA, and a filter setting of 0.10 Hz.

### 4.4. CSF QUIN Determination

QUIN determination was performed using a QUIN ELISA kit (OKCD02284) following the supplier’s instructions (Aviva Systems Biology, San Diego, CA, USA). To remove particulate matter, CSF was centrifuged at 1000× *g* for 10 min, and 50 µL of undiluted CSF samples were used for immunoassays. Optimal color development for TMB substrate was established at 20 min, and absorbance values were determined at 450 nm with a wavelength correction of 540 nm. The QUIN concentration in the CSF samples was determined by linear regression of the relative OD450 value of each sample against a standard curve.

### 4.5. Determination of Interleukin- 4 (IL-4) and Interleukin-6 (IL-6) Concentrations

The concentration of IL-4 and IL-6 in CSF was determined by using the BD^TM^ Cytometric Bead Array (CBA) Human Th1/Th2/Th17 Cytokine kit (BD Biosciences, San Jose, CA, USA; catalog 560484), according to the manufacturer’s instructions. Cytokine standard curves were generated by reconstituting the lyophilized standard and performing serial dilutions. Capture beads specifically for IL-4 and IL-6 were prepared by mixing the corresponding bead population in equal proportions. The working 1× wash buffer was prepared by diluting the supplied 10× stock solution in ultrapure water. For each determination, 50 µL of the phycoerythrin (PE)-conjugated detection reagent was used. Samples were incubated in the dark at room temperature for 3 h. After incubation, 1mL of 1× wash buffer was added, and samples were centrifuged at 200× *g* for 5 min. Supernatants were carefully aspirated, and each pellet was resuspended in 300 µL of 1× wash buffer. Data acquisition was performed using a flow cytometer equipped with a 488 nm excitation laser. At least 300 events per analyte were read. Cytokine concentration was calculated based on a standard curve using the FCAP Array^TM^ software v2. (BD Biosciences). All measurements were performed in accordance with the manufacturer’s quality control recommendations.

### 4.6. Bioinformatic Identification of Genetic Variants Within KP-Related Genes in MS Patients

Publicly available whole-exome sequencing (WES) datasets corresponding to experiments SRP124937 and SRP199159 were retrieved from the Sequence Read Archive (SRA) repository on 13 June 2024 (https://www.ncbi.nlm.nih.gov/sra). Data from experiment SRP124937 corresponded to 3 Moroccan patients affected with MS. WES data from experiment SRP199159 included 3 Italian families: (a) Family A, composed of a non-consanguineous couple where the male individual I-1 was diagnosed with EMP and an EDSS score of 6 at 39 years old, while the female individual I-2 was reported as unaffected. Among their offspring, the male individual II-3 was diagnosed with RRMS and an EDSS score of 1 at 36 years old, and the female individual II-4 was diagnosed with RRMS and an EDSS score of 1.5 at 31 years old; (b) Family B also consisted of a non-consanguineous couple, where the male Individual I-1 was unaffected, and the female individual I-2 was diagnosed with RRMS and an EDSS score of 1.5 at 26 years old. Among their offspring, the male individual II-3 was diagnosed with RRMS and an EDSS score of 1 at 24 years old, while individual II-4 was reported as unaffected; and (c) Family C, which consisted of a non-consanguineous couple, where the male individual I-1 was unaffected, and the female individual I-2 was diagnosed with RRMS and an EDSS score of 3 at 48 years old, and their offspring included a female individual II-3 diagnosed with RRMS and an EDSS score of 4 at 35 years old.

To evaluate WES reads quality and GC content, all SRA-retrieved FASTAQ files were subjected to quality assessment using FastQC (v0.12.1). Then, WES reads were aligned to the reference human genome (GRCh38) using the Burros-Wheeler Aligner (BWA, v0.7.17) using the following parameters: -k 19, -w 100, -d 100, -r 1.5, -c 10000, -A 1, -B 4, -O 6, -E 1, -L 5, -U 17, -T 30; and resulting alignment files were processed and converted into sorted BAM format files using SAMtools (v1.19.2). Then, variant calling was performed using the Genome Analysis Toolkit (GATK v.4.5.0.0) HaplotypeCaller by using the following parameters: sample-diploid 2 and min-base-quality-score 30. To obtain functional interpretation, transcript information, and population allele frequencies of the detected variants, GATK VariantAnnotator and Funcotator bioinformatic tools were used to annotate them. Finally, a targeted analysis focused on identifying genetic variants only in KP-related genes, including *AADAT*, *ACMDS*, *AFMID*, *GOT2*, *HAAO*, *IDO1*, *IDO2*, *KMO*, *KYAT1*, *KYAT3*, *KYNU*, *QPRT*, and *TDO2*, was performed. Variants were called using a minimum variant allele fraction (VAF) > 40 and a sequencing depth > 20×.

### 4.7. Whole-Exome Sequencing of Mexican MS Patients

Genomic DNA (gDNA) was isolated from peripheral blood samples obtained from 3 Mexican MS patients using the Maxwell^®^ 16 Blood DNA Purification Kit (Promega, Madison, WI, USA), according to the manufacturer’s instructions. DNA concentration and purity were assessed using a NanoDrop^TM^ 1000 (Thermo Fisher Scientific, Waltham, MA, USA) and a Qubit fluorometer (Thermo Fisher Scientific, Waltham, MA, USA), respectively. For each sample, 1 µg of gDNA was used to prepare WES libraries using the SOPHiA GENETICS Whole-Exome Sequencing Panel Kit v1 (SOPHiA GENETICS SA, Saint Sulpice, Switzerland) and following the manufacturer’s protocol. Then, library quality and fragment size distribution were verified prior to sequencing. Paired-end sequencing was performed on a NovaSeq 6000X sequencer (Illumina, San Diego, CA, USA). Raw sequencing data were processed and analyzed using the SOPHiA DDM^®^ platform (SOPHiA GENETICS SA, Saint Sulpice, Switzerland), which included aligning WES reads to the genome reference consortium human build 38 (GRCh38). Variant calling and variant annotation were performed using the platform’s already validated pipeline and default parameters that ensure reproducibility.

### 4.8. Statistic

Data were presented as the mean ± SEM. Group comparisons were performed using the Kruskal–Wallis test, followed by Dunn’s test for pairwise comparisons. A *p*-value less than 0.05 was considered statistically significant. Additionally, exploratory analyses of pairwise associations between variables were conducted using Spearman’s correlation coefficient. Analyses were performed using the GraphPad Prism 9.1.0 software (GraphPad, San Diego, CA, USA).

## 5. Conclusions

This exploratory study provides evidence of altered KP metabolism in Mexican naïve patients with MS, particularly during relapse. The identification of variants in KP-related genes further supports the need to investigate potential genetic contributions to this metabolic profile. Given the limited sample size, these findings should be interpreted cautiously. Larger studies are required to confirm these observations and clarify their clinical relevance.

## Figures and Tables

**Table 1:** Demographic and clinical characteristics of Mexican participants. All MS patient subgroups were sampled during clinical relapse.

		HC	Non-MS	NCC	RRMS	PPMS	PMSt
**Sex** *n* (%)	Female	14 (70)	10 (83.3)	1 (20)	18 (72)	10 (62.5)	4 (44.4)
	Male	6 (30)	2 (16.7)	4 (80)	7 (28)	6 (37.5)	5 (55.6)
**Age** (mean ± SEM)		34.8 ± 1.9	47.7 ± 4.1	57.0 ± 6.9	29.7 ± 2	35.1 ± 1.3	29.2 ± 2.6
**Drug naïve** *n* (%)		N/A	N/A	N/A	25 (100)	16 (100)	N/A
**Treatment- receiving patients** *n* (%)	Cyclophosphamide	N/A	N/A	N/A	N/A	N/A	3
	Azathioprine	N/A	N/A	N/A	N/A	N/A	2
	Interferon beta	N/A	N/A	N/A	N/A	N/A	2
	Dimethyl fumarate	N/A	N/A	N/A	N/A	N/A	1
	Fluoxetine	N/A	N/A	N/A	N/A	N/A	1
**EDSS** mean ± SEM		N/A	N/A	N/A	1.9 ± 0.2	5.5 ± 1.2	7.1 ± 0.6

HC: Heatly control; Non-MS: patient with a neurological condition but not Multiple Sclerosis; NCC: Neurocysticercosis; RRMS: relapsing-remitting Multiple Sclerosis; PPMS: Primary Progressive Multiple Sclerosis; PMSt: Progressive Multiple Sclerosis with treatment; EDSS: Expanded Disability Status Scale; N/A: not applicable.

**Table 2:** Genetic variants identified within the sequence of KP-related genes in Multiple Sclerosis (MS) patients.

Gene Name	Transcript Accession ID	Nucleotide Position and Change	Amino Acid Position and Change	Allelic Frequency	Variant Classification
*AFMID*	NM_001010982.5	c.451C>T	p.R151W	0.063853	VB
*KMO*	NM_003679.5	c.809+142C>T	N/A	0.279851	VB
*WARS2*	NM_015836.4	c.119G>A	p.G40D	0.000014	VUS

ID: identifier; VUS: Variant of Uncertain Significance; VB: Benign Variant. Allelic frequency obtained from the gnomAD v.4 database. The position of the referred transcript is specified in the nucleotide column. Transcript accession IDs were obtained from the RefSeq database [29,30].

**Table 3:** Genetic variants identified in genes encoding KP’s enzymes in three Mexican patients with MS.

MS Subtype	Gene Name	Transcript Accession ID	Nucleotide Position and Change	Amino Acid Position and Change	Zygosity
RRMS	*GOT2*	NM_002080.4	c.1037T>G	p.Val346Gly	heterozygous
	*HAAO*	NM_012205.3	c.109A>G	p.Ile37Val	homozygous
	*HAAO*	NM_012205.3	c.124A>T	p.Thr42Ser	homozygous
	*IDO2*	NM_194294.2	c.1077T>A	p.Tyr359*	heterozygous
	*QPRT*	NM_014298.6	c.583A>G	p.Thr195Ala	homozygous
RRMS	*GOT2*	NM_002080.4	c.1037T>G	p.Val346Gly	homozygous
	*HAAO*	NM_012205.3	c.109A>G	p.Ile37Val	homozygous
	*IDO2*	NM_194294.2	c.742C>T	p.Arg248Trp	homozygous
	*QPRT*	NM_014298.6	c.583A>G	p.Thr195Ala	homozygous
PPMS	*GOT2*	NM_002080.4	c.1037T>G	p.Val346Gly	homozygous
	*HAAO*	NM_012205.3	c.109A>G	p.Ile37Val	heterozygous
	*HAAO*	NM_012205.3	c.124A>T	p.Thr42Ser	heterozygous
	*IDO1*	NM_002164.6	c.425C>T	p.Pro142Leu	heterozygous
	*QPRT*	NM_014298.6	c.583A>G	p.Thr195Ala	homozygous

ID: identifier; RRMS: relapsing-remitting Multiple Sclerosis; PPMS: Primary Progressive Multiple Sclerosis; *GOT2*: Glutamicoxaloacetic transaminase 2; *HAAO*: 3-Hydroxyanthranilate 3,4-dioxygenase; *IDO2*: Indoleamine 2,3-Dioxygenase 2; *QPRT:* Quinolinate phosphoribosyltransferase. Transcript accession IDs were obtained from the RefSeq database [29,30].

## Data Availability

The original contributions presented in this study are included in the article. Further inquiries can be directed to the corresponding authors.

## Abbreviations

The following abbreviations are used in this manuscript:

3-HANA	3-Hydroxyanthranilic Acid
3-HK	3-hydroxykynurenine
AA	Anthranilic Acid
BBB	Blood–Brain barrier
CNS	Central Nervous System
CSF	Cerebral Spinal Fluid
EDSS	Expanded Disability Status Scale
GOT2	Glutamicoxaloacetic transaminase 2
HAAO	3-Hydroxyanthranilate 3,4-dioxygenase
IDO1	Indoleamine 2,3-dioxygenase 1
IDO2	Indoleamine 2,3-dioxygenase 2
IL-4	Interleukin-4
IL-6	Interleukin-6
KAT	Kynurenine Aminotransferases
KP	Kynurenine pathway
KYN	L-kynurenine
KYNA	Kynurenic Acid
KYNU	Kynureninase
MS	Multiple Sclerosis
NCC	Neurocysticercosis
NMDA	N-Metyl-D-Aspartate
PMSt	Progressive Multiple Sclerosis under treatment
PPMS	Primary Progressive Multiple Sclerosis
QUIN	Quinolinic Acid
QPRT	Quinolinate phosphoribosyltransferase.
RRMS	Relapsing-remitting Multiple Sclerosis
SRA	Sequence Read Archive
TDO	Tryptophan Dioxygenase
Trp	Tryptophan
VB	Benign Genetic Variants
VUS	Variants of Uncertain Significance
WES	Whole-Exome Sequencing
